# Cancer-secreted AGR2 induces programmed cell death in normal cells

**DOI:** 10.18632/oncotarget.9921

**Published:** 2016-06-08

**Authors:** Elizabeth A. Vitello, Sue-Ing Quek, Heather Kincaid, Thomas Fuchs, Daniel J. Crichton, Pamela Troisch, Alvin Y. Liu

**Affiliations:** ^1^ Department of Urology and Institute for Stem Cell and Regenerative Medicine, University of Washington, Seattle, WA, USA; ^2^ EDRN Informatics Center and NASA Jet Propulsion Laboratory, Pasadena, CA, USA; ^3^ Insititute for Systems Biology, Seattle, WA, USA; ^4^ Present address: Singapore Polytechnic, Center for Biomedical and Life Sciences, Singapore

**Keywords:** AGR2, prostate cancer cell types, prostate stromal cells, programmed cell death, SAT1

## Abstract

Anterior Gradient 2 (AGR2) is a protein expressed in many solid tumor types including prostate, pancreatic, breast and lung. AGR2 functions as a protein disulfide isomerase in the endoplasmic reticulum. However, AGR2 is secreted by cancer cells that overexpress this molecule. Secretion of AGR2 was also found in salamander limb regeneration. Due to its ubiquity, tumor secretion of AGR2 must serve an important role in cancer, yet its molecular function is largely unknown. This study examined the effect of cancer-secreted AGR2 on normal cells. Prostate stromal cells were cultured, and tissue digestion media containing AGR2 prepared from prostate primary cancer 10-076 CP and adenocarcinoma LuCaP 70CR xenograft were added. The control were tissue digestion media containing no AGR2 prepared from benign prostate 10-076 NP and small cell carcinoma LuCaP 145.1 xenograft. In the presence of tumor-secreted AGR2, the stromal cells were found to undergo programmed cell death (PCD) characterized by formation of cellular blebs, cell shrinkage, and DNA fragmentation as seen when the stromal cells were UV irradiated or treated by a pro-apoptotic drug. PCD could be prevented with the addition of the monoclonal AGR2-neutralizing antibody P3A5. DNA microarray analysis of LuCaP 70CR media-treated *vs*. LuCaP 145.1 media-treated cells showed downregulation of the gene *SAT1* as a major change in cells exposed to AGR2. RT-PCR analysis confirmed the array result. *SAT1* encodes spermidine/spermine N^1^-acetyltransferase, which maintains intracellular polyamine levels. Abnormal polyamine metabolism as a result of altered SAT1 activity has an adverse effect on cells through the induction of PCD.

## INTRODUCTION

In the tumor microenvironment, cancer cells interact with surrounding cells in likely different pathways. The interaction between cancer cells and other cells could be mediated by cell-cell contact and diffusible molecules. For example, cell-cell contact mediates expression of prostate-specific antigen in the prostate [1]. Small miRNA in vesicles are transported from producer cell to recipient cell to alter gene expression [2]. In benign prostate glands, three major cell types can be distinguished: CD26^+^ luminal secretory cells, CD104^+^ basal epithelial cells and CD49a^+^ stromal cells [3]. In tumor glands only two major cell types are distinguished: CD26^+^ cancer epithelial cells and CD90^+^ cancer-associated stromal cells [4, 5]. Both CD104^+^ basal cells and CD49a^+^ normal stromal cells are missing. The cause of the disappearance of these cell types is unknown.

In prostate cancer, a majority of primary prostate tumors show elevated expression of anterior gradient 2 (AGR2) [6]. Many prostate cancer metastases in advanced diseases also show high AGR2 expression [7]. AGR2 is a protein disulfide isomerase (PDI) localized to the endoplasmic reticulum (ER) [8]. Expression of AGR2 is found in many solid tumor types including prostate, pancreatic, breast, lung, gastrointestinal and oral [9]. More significantly, cancer cells secrete AGR2 and the protein is found on the cell surface [10-12], whereas normal AGR2^+^ cells do not have expression on the cell surface as the protein is not secreted and is localized to the cell interior [13]. A non-canonical ER retention motif (*C*-terminal KTEL) in AGR2 may be responsible for diverse trafficking of this molecule [14, 15]. Reported functional attributes of AGR2 in cancer include growth promotion and dissemination. Cell lines transfected with AGR2 produced metastasis [16] and gained anchorage-independent growth [17]. AGR2 expression was up-regulated in a gastric cancer cell subline with high metastatic potential for invasion to lymph nodes [18]. Cancer-secreted AGR2 could activate stromal fibroblasts (cancer-associated stromal cells *vs*. normal tissue stromal cells) to promote fibroblast associated cancer invasion of gastric cancer cells [19]. AGR2 could activate ER stress response genes [11], to stimulate cell proliferation of AGR2-negative pancreatic tumor cells, and to enhance drug resistance [10]. Cell surface AGR2 could alter signaling pathways by modulating other cell surface proteins through its disulfide isomerization activity [9]. Other functional aspects for AGR2 reported in the literature include regulation of amphiregulin expression [20], promotion of cell adhesion [21], cancer spread via regulation of cathepsins [11], and cancer cell survival [10].

The cancer-specific secretion of AGR2 indicates an important functional role in cell-cell communication. In salamander limb regeneration, secreted AGR2 was shown to trigger cellular differentiation in responding cells via the receptor Prod1 [22]. In this work, we investigated the effect of cancer-secreted AGR2 on normal prostate stromal cells in culture.

## RESULTS

### Secreted AGR2 in tumor tissue digestion media

**Figure 1:**
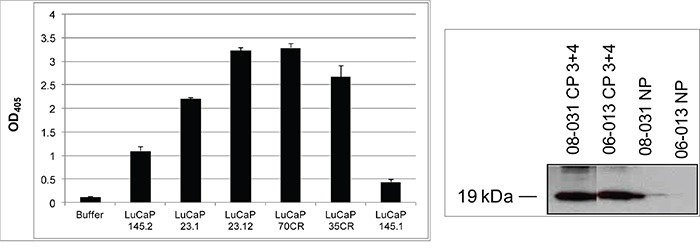
AGR2 levels in tissue digestion media Left panel shows the relative protein levels (OD_405_ readings on the *y*-axis) in LuCaP digestion media measured by ELISA. Right panel shows Western blotting of tissue digestion media of CP (both Gleason 3+4) *vs*. NP. The molecular weight of AGR2 was estimated at 19 kDa.

**Figure 2:**
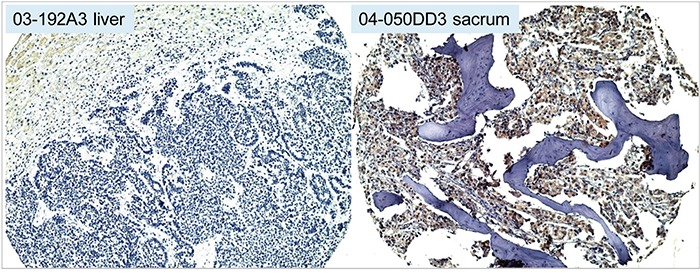
Prostate cancer metastasis expression of AGR2 Immunohistochemistry shows no AGR2 expression by small cell carcinoma in the liver (with faint staining in the liver cells, upper left of left photomicrograph) and high AGR2 expression by the adenocarcinoma in bone (brown stain, right photomicrograph).

The LuCaP prostate cancer xenograft lines [23] established from primary neoplasm and metastases were either AGR2^+^ adenocarcinoma or AGR2^−^ small cell carcinoma [7]. The tumors were harvested from mice and digested by collagenase. The cell-free media supernatant contained proteins secreted or released by the human tumor cells [24]. AGR2 in the cell-free digestion media was measured by ELISA (Figure 1). All the adenocarcinoma media tested were positive for AGR2 as shown for LuCaP 23.1, LuCaP 23.12, LuCaP 70CR, and LuCaP 35CR (CR = castration resistant). In contrast, the small cell carcinoma LuCaP 145.1 media was near buffer background. A sister line LuCaP 145.2 was positive for AGR2 but at a lower level than those of the adenocarcinoma lines. It could represent a small cell carcinoma with some AGR2 expression or a mixed small cell carcinoma/adenocarcinoma. AGR2 concentration ranged from >100 pg/ml for LuCaP 70CR to <2pg/ml for LuCaP 145.1. For the following culture experiments, media of AGR2^+^ LuCaP 70CR [established from an autopsied liver metastasis, expressed wild type androgen receptor (AR) and moderate level of prostate-specific antigen (PSA)] and AGR2^−^ LuCaP 145.1 (established from a liver metastasis, expressed no AR and PSA) were used. Immunostaining of adenocarcinoma (in a bone metastasis) and small cell carcinoma (in a liver metastasis) obtained from autopsies showed the difference in AGR2 expression between these two cancer types (Figure 2). In the small cell carcinoma section, liver cells adjacent to the tumor mass were moderately stained for AGR2 expression whereas the tumor cells were completely unstained (note that AGR2 in normal cells such as bladder urothelial cells is not secreted [13]). Surgically resected primary tumors were also processed for tissue digestion [1]. Benign tissue (NP) media contained no AGR2 in contrast to tumor tissue (CP) media. Western blot data in Figure 1 show the presence of secreted AGR2 of 19 kDa in size in two CP media and its absence in the corresponding matched NP media. AGR2 was found to be the most abundant secreted proteins of <20 (besides prostate-specific antigen, prostatic acid phosphatase) produced by prostate cancer cells [4, 12]. Media of sample 10-076 CP and the matched 10-076 NP were used for culture because of their large volume. Immunostaining of prostate primary tumors was reported previously, where most of the cases stained were positive for AGR2 [6].

### Induction of cellular abnormalities by AGR2 on NP stromal cells

**Figure 3:**
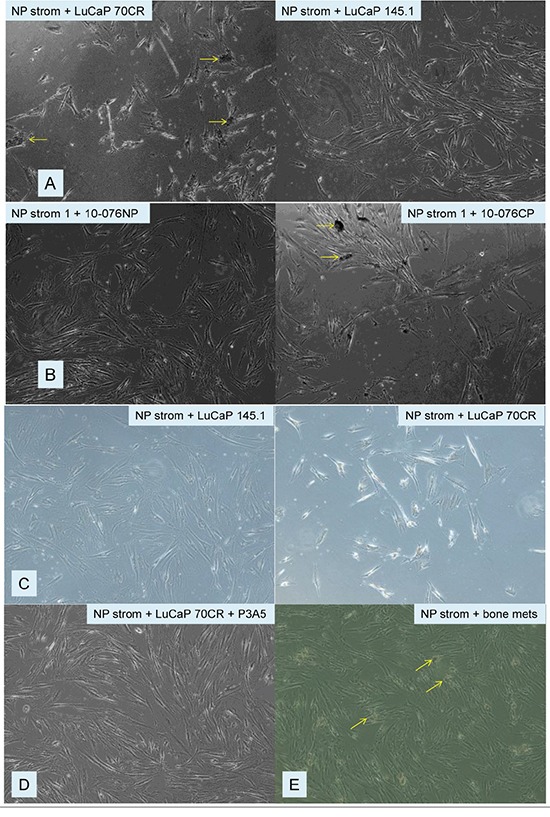
Effect of AGR2-containing media on NP strom cells **A.** Cellular blebs (yellow arrows) are prominent in the NP strom + LuCaP 70CR culture; no blebs are seen in the NP strom + LuCaP 145.1 culture. **B.** Blebs (yellow arrows) are seen in the NP strom + 10-076 CP culture *vs*. the NP strom + 10-076 NP culture. **C.** The abnormal cells in NP strom + LuCaP 70CR appear with a bright halo, and are smaller in length compared to the cells in NP strom + LuCaP 145.1. **D.** Blebbing was prevented by the addition of anti-AGR2 P3A5. **E.** Blebbing is also seen in the culture with metastasis digestion media containing AGR2.

Normal prostate stromal (NP strom) cells were prepared from benign tissue specimens and cultured in serum-supplemented media [25]. Near confluence, half of the media was replaced by the various tissue digestion media described above. Low passage NP strom cells incubated with AGR2^+^ LuCaP 70CR digestion media showed cellular blebbing or clustered protrusions of the plasma membrane compared to those with AGR2^−^ LuCaP 145.1 media (Figure 3A). These abnormalities were also observed for NP strom cells with AGR2^+^ 10-076 CP media compared to those with AGR2^−^ 10-076 NP media (Figure 3B). Cell shrinkage was evident with cells showing a “bright halo” around the abnormalities (Figure 3C). Such a visual change was also reported for oral fibroblasts undergoing apoptosis [26]. As could be observed, nearly all the cells in the AGR2-containing cultures showed these morphological changes. After ~24 h, there were no viable cells in the LuCaP 70CR and 10-076 CP cultures while cells in the LuCaP 145.1 and 10-076 NP cultures remained healthy. AGR2 was a major common molecule in the media of LuCaP 70CR and 10-076 CP as indicated by strong immunostaining of the respective tumor tissue [6, 7]. CP media differed chiefly from LuCaP media in the presence of molecules secreted by the cancer-associated stromal cells and other minor cell types of the primary tumor, which were absent in the xenografts. Xenograft tumors also contained infiltrating mouse cells. That AGR2 was the causative factor was shown by the addition of anti-AGR2 monoclonal P3A5 in the culture. P3A5 was developed for use in AGR2 ELISA [12]. The effect of AGR2 was neutralized, and the cellular abnormalities were not seen (Figure 3D). Other AGR2^+^ adenocarcinoma LuCaP media (not shown) as well as collagenase digestion media of metastasis samples obtained directly from donor autopsies (Figure 3E) produced the same result. AGR2 levels in these other tissue media were measured by ELISA as documented previously [12]. The exogenously added bacterial collagenase could not produce the effect since it was present in all media preparations.

### Induction of DNA fragmentation in treated cells by AGR2

**Figure 4:**
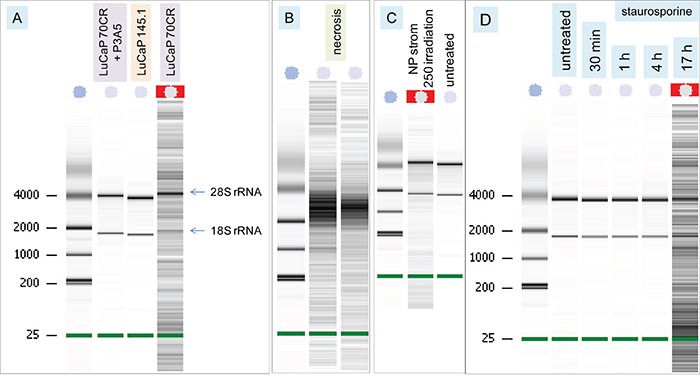
DNA fragmentation of AGR2-treated cells **A.** Bioanalyzer traces show the nucleic acid patterns of cultures listed on the top. The ribosomal RNA species are indicated. The left lane contains size standards. **B.** Traces show the patterns of necrotic NP strom cells. **C.** Traces compare the patterns of UV-irradiated (label 250 denotes 25 mJ/cm^2^) NP strom cells and untreated cells. **D.** Traces show the time course of staurosporine treatment.

RNA was isolated from the treated NP strom cells and analyzed by Agilent Bioanalyzer. The Bioanalyzer profile of untreated NP strom cells displayed intact 28S and 18S ribosomal RNA (rRNA) and faint traces of mRNA. However, the isolated RNA from LuCaP 70CR-treated NP strom cells showed, in addition, low molecular weight DNA fragments but that from LuCaP 145.1-treated cells did not (Figure 4A). Although the RNA isolation kit was designed for RNA purification, any small DNA present in the sample could also be captured [27]. Exogenously added RNA would prevent binding of DNA in the sample. The presence of intact 28S and 18S rRNA indicated no RNA degradation in the affected NP strom cells. DNA degradation without RNA degradation was diagnostic of programmed cell death (PCD)/apoptosis [28], as was formation of cellular abnormalities. In contrast, cell necrosis as caused by electroporation of NP strom cells led to both DNA and RNA degradation with no intact rRNA bands (Figure 4B). No DNA fragmentation was seen with added P3A5 antibody in the culture of LuCaP 70CR-treated NP strom cells (Figure 4A, lane 2). As positive controls of PCD, NP strom cells were treated by UV-irradiation and the pro-apoptotic drug staurosporine. DNA fragmentation with intact rRNA was found in irradiated NP strom cells (Figure 4C). Addition of staurosporine produced cell morphology changes within an hour while DNA fragmentation was found at >16 h (Figure 4D).

### Down-regulation of *SAT1* in cells cultured in the presence of AGR2

**Figure 5:**
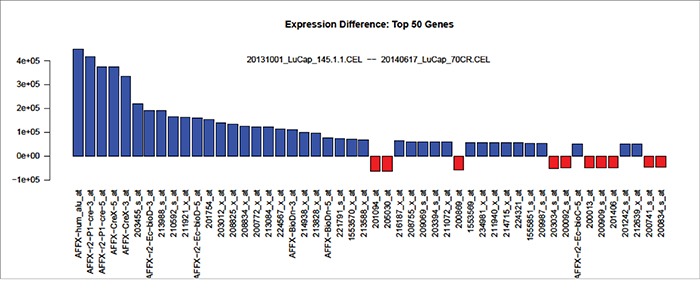
Gene expression changes induced by AGR2-containing media Blue bars indicate genes with higher expression in NP strom cells incubated with LuCaP 145.1 media; red bars indicate genes with higher expression in cells incubated with LuCaP 70CR media. Ten of the 50 entries listed are Affymetrix control probesets.

The RNA (without DNA fragments, i.e., preceding DNA fragmentation) from LuCaP 70CR- and LuCaP 145.1-treated NP strom cells was analyzed by Affymetrix DNA microarrays for differential gene expression. The result showed that only a small number of differentially expressed genes (<30 of 54,675) were detected between the two. A major difference was the down-regulation of spermidine/spermine N^1^-acetyltransferase (SAT1) in AGR2-treated NP strom cells with all three SAT1 probesets at the top of the gene listing display showing a 2e+05 fold difference in Figure 5. The differentially expressed genes (blue *vs*. red) encoded by the probesets in the figure are listed in Supplementary Table 1. Other down-regulated genes included ribosomal subunit protein RPL23a, COX6C, COX2, and prothymosin-like α. The latter was reported in the literature to possess an anti-apoptotic function [29]. In contrast, not many genes were affected in cells exposed to AGR2^−^ LuCaP 145.1 media.

### RT-PCR verification of the SAT1 array result

**Figure 6:**
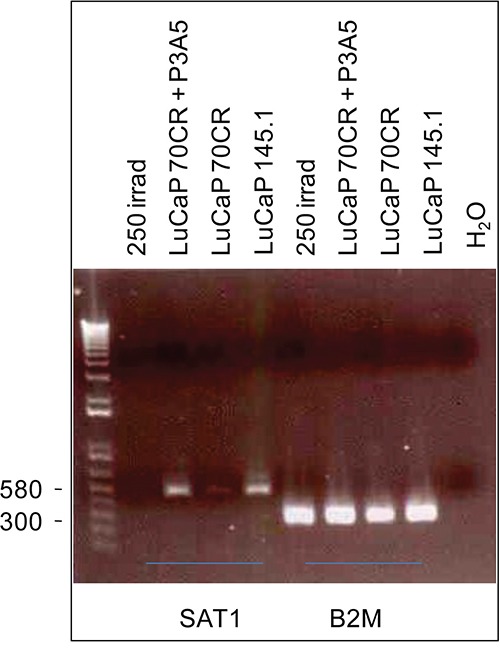
Differential expression of *SAT1* *SAT1* expression levels in the cell cultures listed on the top are represented by the PCR product band intensity. The house-keeping gene B2M bands show equal loading.

Down-regulation of *SAT1* in LuCaP 70CR-treated NP strom cells was verified by RT-PCR analysis. The SAT1 PCR product showed a lower band intensity in NP strom + LuCaP 70CR than NP strom + LuCaP 145.1 (Figure 6) in agreement with the quantitative (signal intensity values) difference found by the array analysis. B2M beta2-microglobulin served as the reaction control, whose PCR band showed similar intensity in all the RNA tested. The data also showed that *SAT1* expression was not affected in NP strom + LuCaP 70CR + anti-AGR2 P3A5. *SAT1* expression was also down-regulated in UV-irradiated NP strom cells.

## DISCUSSION

In normal cells, AGR2 functions as a PDI in the ER. In cancer cells, AGR2 is also secreted. Cancer secretion could be due to saturation of the ER receptor sites as AGR2 is over-expressed in cancer cells. The functional role of cancer-secreted AGR2 on neighboring cells is unknown. Here, we showed that secreted AGR2 could induce formation of cellular protrusions in prostate stromal cells in culture, which is due to the loss of cytoskeletal integrity. This was followed by chromosomal DNA fragmentation. These features are well-known characteristics PCD [28, 30, 31]. In the culture experiments, the source of AGR2 was prostate primary tumors, prostate adenocarcinoma xenografts, as well as prostate cancer metastases. Although we cannot definitively rule out other molecules present in the PCD-inducing tissue digestion media but absent in media of benign prostate and a small cell carcinoma xenograft, AGR2 is a common high abundance molecule among these sources. Furthermore, anti-AGR2 P3A5 antibody could prevent cellular abnormalities and DNA breakdown when added to the AGR2^+^ culture media. We are currently devising a protocol to purify AGR2 from tissue culture media of prostate cancer cell lines PC3 or CL1 [12] for use in future experiments. Purified AGR2 will also allow us to identify any structural differences in the secreted form of the protein *vs*. the ER-localized form.

We used prostate stromal cells first to study the effect of extracellular AGR2 because these normal cells are readily obtainable in sufficient amounts after culture from donated surgical specimens. In prostate tumor glands in vivo, the stromal cells adjacent to the AGR2^+^ cancer cells are CD90^+^ cancer-associated (CP) stromal cells, which differ from NP stromal cells in overall gene expression [5]. At least a 20-cell length separates the tumor epithelial cells from NP stromal cells of adjacent benign glands [32]. Whether the CP stromal cells, like NP stromal cells, are susceptible to AGR2-induced PCD in culture remains to be determined, which would require cell isolation from a sizable surgical specimen by using CD90 antibodies. PCD induced by AGR2 could also explain the absence of CD104^+^ basal epithelial cells in prostate tumor glands [32], which is a diagnostic criterion for prostate cancer. Additionally, cancer-secreted AGR2 could facilitate tumor spread by inducing PCD in endothelial cells of the vessel lining, making blood vessels leaky to allow access by the cancer cells [33].

In salamander limb regeneration, secreted AGR2 interacts with a cell surface receptor on adjacent cells identified as Prod1. The putative human homolog of Prod1 is CD59. Whether CD59 is responsible for transducing the effect of AGR2 is at present unknown. Both NP strom and prostate basal cells are immunostained by anti-CD59 [34]. Cells without CD59 might, therefore, be resistant to the effect of AGR2. Another possible future study could determine the effect of secreted AGR2 on stem cells such as the induced pluripotent stem cells generated from reprogramming of prostate stromal cells [35], given its function in organ regeneration.

The immediate effect of AGR2 appears to be the down-regulation of *SAT1*, which was also observed in UV-irradiated cells. PCD can be triggered by many stimuli, both physiological and non-physiological. The down-regulation of SAT1 by AGR2 treatment and irradiation, which in turn affects intracellular polyamine levels, could be the trigger to initiate the PCD response. SAT1 is a key enzyme in the maintenance of intracellular polyamine levels through acetylation of its substrates for conversion to putrescine [36]. Polyamines have a global effect on gene transcription, and perturb action of ion channels to cause cell shrinkage as seen in PCD [37]. One of the ion channel genes affected in LuCaP 70CR-treated cells was *ATP1B1*, the product of which functions in maintaining electrochemical gradients of Na and K ions across the plasma membrane. Thus, an abnormal level of polyamines as a result of lower SAT1 activity produces an adverse effect on cells leading to cell death [38]. A link between tumor resistance to ionizing radiation and overexpression of *SAT1* was recently reported in brain cancer [39]. This finding provided support for *SAT1* expression loss induced by irradiation. Other research data linked depletion of intracellular polyamines through increased SAT1 activity to cell death as well [40, 41]. For example, in EBV-positive lymphoma cell clones SAT1 activity is lowered compared with EBV-negative cell clones, which promoted cell growth [42].

In summary, secretion and cell surface expression of AGR2 are specific to many solid tumors. Cancer-secreted AGR2 induces cell death in normal (prostate stromal) cells with down-regulation of *SAT1*, which is involved in polyamine metabolism. In prostate cancer metastasis, the tumor-derived AGR2 could contribute significantly to organ failure with the destruction of normal cells. The PCD-inducing property of AGR2 may be responsible for the irreversible loss of body mass in cancer cachexia. Thus, minimizing its deleterious effect by the use of anti-AGR2 agents may prove clinically beneficial.

## MATERIALS AND METHODS

### AGR2-containing tissue digestion media preparations

Collagenase tissue digestion media was used to determine the effect of cancer-secreted AGR2. This media was prepared from enzymatic digestion of tissue specimens - surgically resected prostates and LuCaP prostate cancer xenografts (established from tumor specimens obtained from patients implanted in immune-compromised mice) - in RPMI1640 supplemented with 5% fetal bovine serum (FBS) and gentamycin sulfate [1]. Three ml media and collagenase were added to 0.1 g minced tissue specimens. After tissue digestion, ~1 h for xenografts to overnight for surgical specimens, the media was diluted by an equal volume of Hanks balanced salt solution (HBSS), passed through a cell strainer, and centrifuged. The cell-free supernatant contained secreted molecules such as AGR2. Since tissue digestion was carried out similarly between 10-076 CP and 10-076 NP (control), LuCaP 70CR and LuCaP 145.1 (control), the media of all four would be “contaminated” by more or less the same cytoplasmic proteins. The 10-076 CP tumor tissue sample was carefully microdissected by our pathologist colleague to ensure minimal “contamination” of benign tissue. The collected tissue was processed immediately so necrosis was minimal. The amount of AGR2 in these media preparations was measured by our sandwich ELISA [12] or detected by Western blotting. In ELISA, recombinant AGR2 (GenWay Biotech, San Diego, CA) was used to generate a standard curve for calibration. BD Falcon plates (Fisher Scientific, Pittsburgh, PA) were coated with 1:1,000 purified anti-AGR2 monoclonal P1G4 (IgG1) in phosphate-buffered saline (PBS), rinsed with PBS-0.05% Tween, and blocked with 1% heat-denatured bovine serum albumin. The media samples were added for incubation at 4° overnight. For detection, 1:1,000 purified anti-AGR2 monoclonal P3A5 (IgG2a) was used, followed by HRP-conjugated anti-mouse IgG2a. The chromogen was 2,2′-azinobis[3-ethylbenzothiazoline-6-sulfonic acid]-diammonium salt (KPL/Fisher, Belgium), and the reaction was scored at λ= 405 nm. The cell-free media supernatants were stored frozen, and no significant loss of AGR2 was detected over several years. In Western blot, gel electrophoresis was carried out under reducing conditions, and AGR2 was detected by P1G4 (1:19) followed by secondary anti-mouse IgG1 (1:1,000). Matched digestion media samples of 06-013 NP and 06-013 CP (NP = benign prostate, CP = prostate cancer) Gleason score 3+4, 08-031 NP and 08-031 CP Gleason score 3+4 were compared for the level of AGR2. Equal gel loading of the samples was verified by Western blot of prostate-specific antigen. The Western blot data confirmed immunohistochemistry of benign prostate tissue showing no AGR2 expression.

The media used in this study were prepared from primary prostate cancer AGR2-positive 10-076 CP (Gleason score 5+4+3, tumor volume of 5.5 cc) and AGR2-negative 10-076 NP matched benign (i.e., distal to the tumor area); AGR2-positive adenocarcinoma xenograft LuCaP 70CR and AGR2-negative small cell carcinoma xenograft LuCaP 145.1.

### Prostate stromal cell cultures

Normal prostate stromal cells (NP strom) were cultured from resected fresh tissue pieces [25]. The tissue was minced and seeded on plastic plates with RPMI1640 + 10% FBS, 37°C. When outgrowths from the tissue pieces were evident after one week, the cells were trypsinized and passaged. Due to the lower plating efficiency of epithelial cells, these cells were eliminated after a few passages. In culture, NP strom cells displayed a fibroblastic-like morphology. Previously, DNA microarray analysis of cultured NP strom cells derived from multiple patient specimens showed a similar gene expression to each other [43]. Cells obtained from specimen 14-089 NP were used in the study. NP strom cells at low passage number were stored frozen and thawed as needed.

### Treatment of NP strom cells

NP strom cells were cultured in 6-well BD Falcon plates (Fisher Scientific). At near confluence, half of the culture media was replaced by the various tissue digestion media. At different times of incubation, the cells were washed with HBSS and photographed under magnification. The treated cells were then trypsinized for RNA isolation. Small DNA molecules, if present, could be isolated by the RNA isolation kit (Ambion Life Technologies, Carlsbad, CA) used. The RNA preparations were analyzed by Bioanalyzer (Agilent Technologies, Santa Clara, CA). DNA microarrays - Affymetrix HU133 Plus 2.0 GeneChips - were used to analyze for differential gene expression of NP strom cells treated by LuCaP 70CR *vs*. LuCaP 145.1 media. Monoclonal AGR2 antibody P3A5 was added (50 μl of 3.6 mg/ml per well) to determine if the effect of AGR2 could be neutralized. AGR2 specificity of P3A5 was demonstrated by immunoprecipitation of AGR2 in AGR2-secreting prostate cancer cell culture media, and its use in sandwich ELISA to measure urinary AGR2 [12]. Bacterially made recombinant AGR2 was not used because mammalian proteins in bacteria are not folded correctly and lack proper post-translational modifications. In addition, NP strom cells were irradiated at 25 mjoule/cm^2^ in a Ultra-Violet Crosslinker (Cole Palmer, Vernon Hills, IL) or treated with staurosporine (Sigma, St. Louis, MO) at 1 μM. Cells were photographed and RNA was isolated from different time points. Cell necrosis was induced by electroporation using an AMAXA nucleoporator (Lonza, Basel, Switzerland).

### Gene expression analyses

Differential gene expression analysis from array datasets was described in our previous publications [3, 44]. The obtained array CEL files were loaded into GeneSpring 7.2 (Agilent Technologies), which used an analysis algorithm of the open source Bioconductor project [45]. Reverse transcriptase polymerase chain reaction (RT-PCR) was used to confirm the array result. Oligonucleotide primers were SAT1-5 GACTGCAGTGACATACTGCGG and SAT1-3 ACAGCAGCACTCCTCACTCCT; B2M-5 GGCTATCCAGCGTACTCCAAAGATTC and B2M-3 GTCTCGATCCCACTTAACTATCTTGGGC. The expected product size for SAT1 was 492 bp and that for B2M 297 bp. The cycling parameters were 94°, 30 s; 57°, 30 s; 72°, 1 min for 35 cycles. The reaction products were resolved by agarose gel electrophoresis.

## SUPPLEMENTARY MATERIALS TABLES




