# Supplementary material for: Cancer-secreted AGR2 induces programmed cell death in normal cells
Source: Oncotarget. 2016 Jun 8;7(31):49425–34. doi: 10.18632/oncotarget.9921 (PMC5226518; doi:10.18632/oncotarget.9921)
Supplement: Supplementary file 1 [file oncotarget-07-49425-s001.pdf]

Cancer-secreted AGR2 induces programmed cell death in normal cells

SUPPLEMENTARY TABLE

Supplementary Table S1: Affymetrix probesets and gene names. Highlighted entries are genes down-regulated in NP strom + LuCaP 145.1

See Supplementary File 1
